# Anterior-Segment Swept-Source Ocular Coherence Tomography and Scheimpflug Imaging Agreement for Keratometry and Pupil Measurements in Healthy Eyes

**DOI:** 10.3390/jcm10245789

**Published:** 2021-12-10

**Authors:** Francisco Pérez-Bartolomé, Carlos Rocha-De-Lossada, José-María Sánchez-González, Silvia Feu-Basilio, Josep Torras-Sanvicens, Jorge Peraza-Nieves

**Affiliations:** 1 Department of Ophthalmology, Hospital Universitario Puerta de Hierro, 28222 Majadahonda, Spain; franciscoperezbartolome@gmail.com; 2 Department of Ophthalmology, Clinic Institute of Ophthalmology, Hospital Clinic of Barcelona, University of Barcelona, 08036 Barcelona, Spain; silviafeub@gmail.com (S.F.-B.); jts29206@gmail.com (J.T.-S.); jorge.peraza.nieves@gmail.com (J.P.-N.); 3 Department of Ophthalmology (Qvision), Vithas Virgen del Mar Hospital, 04120 Almería, Spain; carlosrochadelossada5@gmail.com; 4 Department of Ophthalmology, Hospital Virgen de Las Nieves, 18014 Granada, Spain; 5 Department of Ophthalmology, Ceuta Medical Center, 51001 Ceuta, Spain; 6 Department of Physics of Condensed Matter, Optics Area, Vision Science Research Group (CIVIUS), Pharmacy Faculty, University of Seville, 41012 Sevilla, Spain

**Keywords:** anterior-segment swept-source, ocular coherence tomography, Scheimpflug imaging, agreement analysis, pupil measurements

## Abstract

This study examines agreement between the devices Anterion^®^ and Pentacam HR^®^ used for corneal and pupil measurements in healthy eyes. The parameters compared between the two devices were: anterior Km (D), anterior K2 (D), anterior K1 (D), anterior K1 axis (°), anterior astigmatism (D), anterior K max (D), posterior Km (D), posterior K2 (D), posterior K1 (D), posterior K1 axis (°), posterior astigmatism (D), CCT (µm), thinnest point thickness (µm), thinnest point X-coordinate (mm), thinnest point Y-coordinate (mm), pupil diameter (mm), pupil center-corneal vertex distance (mm) (angle kappa), pupil centroid angle (°), pupil centroid X-coordinate (mm), and pupil centroid Y-coordinate (mm). The Student’s *t* test for independent samples identified significant differences (*p* < 0.005) between devices for the measurements anterior and posterior flat K axis, posterior flat K, steep K, and mean K. For these last three measurements, although significant, none of the differences were clinically relevant. Corneal power and thickness measurements except Kf axis showed excellent agreement between Anterion and Pentacam. In a clinical setting we would not recommend the interchangeable use of Pentacam and Anterion for measurement of pupil parameters.

## 1. Introduction

Precise measurement of both corneal power and pupil diameter has progressively gained importance in parallel with the development of cataract and refractive surgery procedures [1]. Central corneal thickness (CCT), and mean (Km), flat (Kf) and steep (Ks) keratometry, along with pupil diameter (PD), are main determining factors for excimer laser ablation and/or multifocal intraocular lens (MIOL) implantation [2,3]. One of the devices most used in clinical practice for these measurements is the Pentacam^®^ HR (OCULUS Optikgeräte GmbH), a rotating Scheimpflug camera system designed to visualize the anterior segment that is able to measure corneal topography, elevation of the anterior and posterior corneal surface, corneal thickness, and anterior chamber angle [1,4].

Based on a completely different technology, the Anterion^®^ (Heidelberg Engineering, Heidelberg, Germany) is a multimodal imaging device introduced in 2019 [5]. Through anterior segment swept-source optical coherence tomography (OCT), it can be used to measures axial length for ocular biometry and can also be used to image the anterior segment. The cornea display of the device offers a wide range of anterior segment parameters such as anterior and posterior keratometry, central corneal thickness, elevation measures, and pupil size.

For anterior segment measurements, studies have shown variable repeatability and reproducibility between Pentacam^®^ HR and Anterion, and between Anterion and other OCT-based devices, such as CASIA (Tomey Corporation, Nagoya, Japan) [6,7,8,9,10]. With regard to the latest research, Brunner et al. [11] investigated repeatability coefficients and limits of agreement comparing Pentacam HR, Orbscan IIz, and Tomey Casia SS-1000 measurements. However, to the best of our knowledge, no investigation has compared pupil measurements provided by Anterion and Pentacam HR. The aim of the present study was to determine agreement between these two devices for both keratometry and pupil measurements in healthy individuals.

## 2. Materials and Methods

This was a prospective cross-sectional study. The study protocol was approved by the Ethics committee of the Hospital Clinic de Barcelona (HCB/2021/0388) and adhered to the principles of the Declaration of Helsinki. Informed consent was obtained from each participant at the time of enrollment.

### 2.1. Study Population

Participants were 56 healthy volunteers recruited among hospital staff and their relatives. While examinations were performed in both eyes, only data for one eye were analyzed. Inclusion criteria were age ≥18 years, no history of ocular surgery or contact lens use, and no corneal disease, ocular trauma, or systemic collagen disease.

### 2.2. Devices

The Anterion device uses swept-source AS-OCT technology with a 1300 nm wavelength light source, and speed of 50,000 A-scans/second. This device images the anterior segment of the eye at an axial depth of 14 mm, lateral width of 16.5 mm, in-tissue axial resolution less than 10 μm and lateral resolution 30–45 mm [12]. All measurements are assisted by eye-tracking technology centered on the corneal vertex [12].

The Pentacam HR uses a rotating Scheimpflug camera which takes 100 images of 500 measurement points on the anterior and posterior corneal surfaces over 180 degrees of rotation [13]. Elevation data from all these images are combined to form a three-dimensional reconstruction of corneal shape within the same diameter optic zones as Anterion.

### 2.3. Examination Protocol

All patients underwent a complete ophthalmic examination, including distance-corrected visual acuity measurement, anterior segment bio-microscopy and fundus examination. After three minutes of dark adaptation, the same experienced ophthalmologist performed a single scan of both eyes, first using the cornea mode of Anterion and then the Pentacam HR in standard light conditions.

Subjects were instructed to maintain the imaged eye on the central fixation target built into each device. Only scans of optimal quality indicated as over 95% by the Pentacam HR and as “green” by the Anterion were included in the analysis. Subjects with eye conditions that could affect fixation and the quality of data acquisition such as corneal disease, cataract or any maculopathy were excluded. Scans obtained in subjects with suspected or confirmed keratoconus (Kmax index >48 D, irregular astigmatism, infero-temporal displacement of the thinnest corneal point or an abnormal Belin-Ambrosio Pentacam analysis) or another corneal ectasia were also discarded.

Data from the Anterion wavefront analysis were exported to an excel sheet. From each device, 20 parameters were automatically exported: 11 related to both anterior and posterior corneal surface measurements: Kf, Km, Ks, Kf axis and astigmatism; four related to corneal thickness: CCT, thinnest point and its location; five related to pupil measurements: PD, center from visual axis (angle kappa), pupil center angle (between pupil center plane and corneal surface) and position with vectors X and Y. The same parameters of the Pentacam were manually entered in the same excel sheet used for the Anterion data. Both eyes were examined, but only right eye data were included. If right eye was not suitable for inclusion, left eye data was included.

### 2.4. Statistical Analysis

All data were analyzed using SPSS (version 22.0, IBM Corp., Armonk, NY, USA) and Excel software (2016, Microsoft Corp., Redmond, WA, USA). Results are expressed as the mean ± standard deviation (SD). Significance was set at *p* < 0.05. The Shapiro-Wilk test was used to confirm the normal distribution of data (*p* > 0.05). Initially, the Student’s *t* test for independent samples was used to identify differences between devices.

Inter-device agreement for each corneal aberration parameter was assessed through mean differences, paired t-test for mean differences, and 95% confidence intervals and limits of agreement (LoA). Bland-Altman plots were also constructed using the LoAs to compare both devices. Sample size calculation was based on recent studies that have analyzed agreement between AS-OCT and Scheimpflug devices [6,7]. For a 2-sided level of significance (α) at 0.05 and power (β) of 80%, the sample-size calculation indicated that a minimum of 50 participants was required to detect a difference of 0.1 µm in all measurements.

## 3. Results

The 56 subjects recruited were 32 women (57.14%) and 24 men (42.85%) of mean age 52.35 years ± 20.2 SD (21–78). Table 1 provides a descriptive analysis of the study sample. The Student’s *t* test for independent samples identified significant differences (*p* < 0.05) between devices for the measurements of anterior and posterior flat K axis, posterior flat K, steep K, and mean K. For these last three measurements, although significant, none of the differences were clinically relevant.

**Figure 1:**
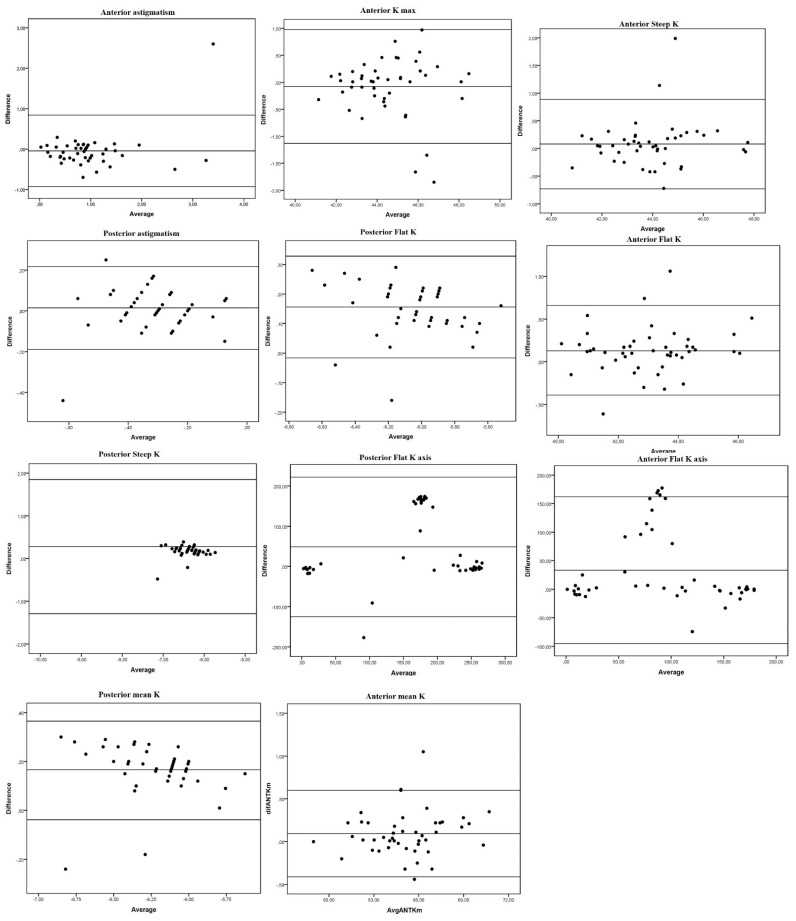
Bland-Altman plots of mean differences against averages of anterior and posterior corneal surface keratometry readings. Mean, lower and upper limits of agreement (±1.96 SD, standard deviation). Left: anterior vertical coma. Right: total vertical coma.

**Figure 2:**
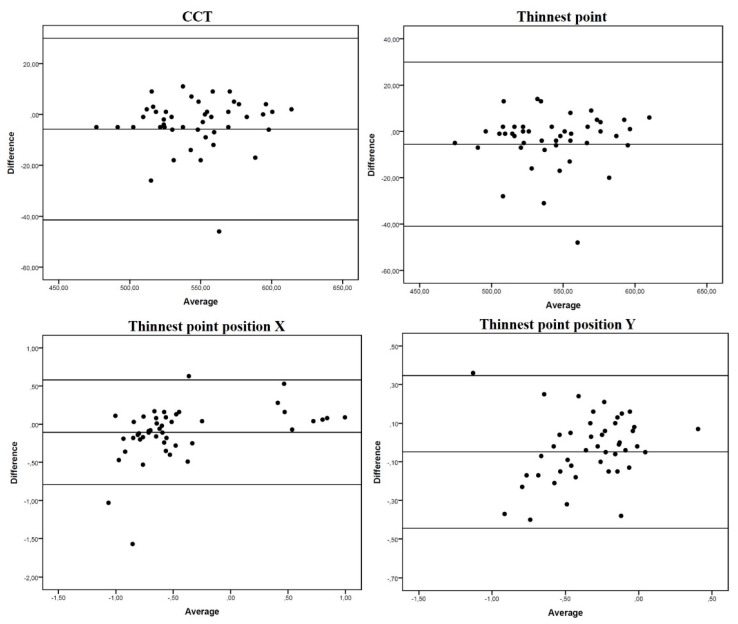
Bland-Altman plots of mean differences against averages of pachy-metric measurements. Mean, lower and upper limits of agreement (±1.96 SD, standard deviation).

**Figure 3:**
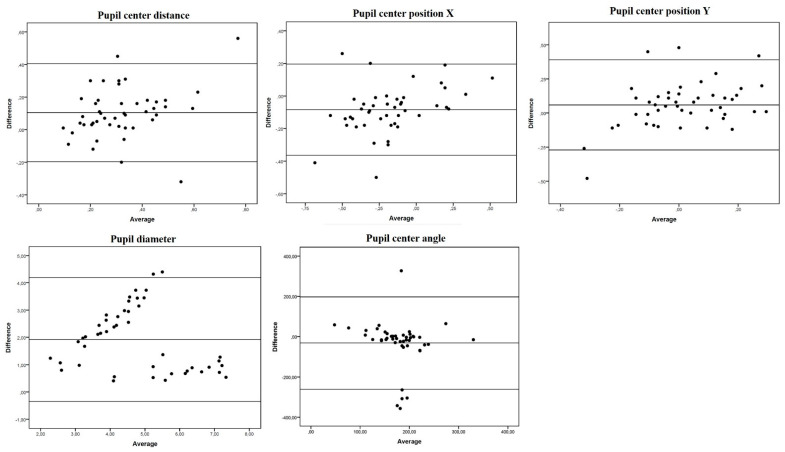
Bland-Altman plots of mean differences against averages of pupil measurements. Mean, lower and upper limits of agreement (±1.96 SD, standard deviation).

Overall, mean differences were small, with narrow limits of agreement (LOA) (Table 2, Figure 1 and Figure 2). Although the paired t-test revealed significant differences for all the parameters studied (except posterior steep K, *p* = 0.051), differences in absolute values were generally close to 0. Thus, overall, good agreement was found between the Anterion and Pentacam measures. Nonetheless, we found some exceptions. Both anterior and posterior Kf axis showed poor agreement between devices (large mean difference with wide LOA) (Table 2 and Figure 1). For pupil measurements, positions in the X and Y axis were highly concordant, but PD, pupil center from visual axis (angle kappa) and pupil center angle were not (Table 2 and Figure 3).

## 4. Discussion

Anterior segment measurements are used as cut-offs for patient and ocular surgical method selection. In this study, excellent agreement was observed between both devices for kerato-metric and pachy-metric measurements, while pupil parameter measurements usually differed significantly. PD has been described as a main factor in indicating refractive surgery [2,3]. In addition, scotopic larger pupils can overlap ablation optic zones and optic diameters of MIOL resulting in poor night vision and halos [14]. PD is influenced by the light of the media and undergoes size reduction with accommodation [4]. Therefore, it is not clear whether the Pentacam is able to provide clinically useful information prior to refractive surgery as it uses a 450-nm visible blue light-emitting diode which can induce miosis. In effect, here we recorded a smaller PD with the Pentacam (3.82 ± 1.6 vs. 5.74 ± 1.34) (Table 1). Being based on OCT technology, Anterion does not induce such intense miosis and could theoretically be a better method to measure mesopic PD. Güçlü et al. [15] reported that IOL-Master 700, another OCT-based biometer, is a useful device that is interchangeable with Pentacam for keratometry values and axis, but not for white-to-white distance (WTW), anterior chamber depth (ACD), and CCT. In their study, mean PD determined in healthy eyes was 6.4 ± 1.4 mm with IOL-Master 700 and 4.5 ± 1.3 mm with Pentacam [15]. In our study, slightly smaller PDs were recorded but with the same trend, probably because we included older subjects. Our Bland-Altman plots indicated great disparity with wide LOAs (−0.35–4.2) for our PD measurements (Figure 3). Therefore, we would not recommend the interchangeable use of Pentacam and Anterion for PD measurements.

Recently, besides PD, much attention has been paid to other causes of disturbing ocular symptoms after MIOL implantation, such as glare and halos. Studies so far have shown that any large deviation between the optical center or visual axis and the pupillary axis of the MIOL can lead to higher-order aberrations postoperatively, compromising visual quality [16,17,18,19]. Both Pentacam and Anterior are able to determine the position of the pupil center through two vectors (X and Y), the angle kappa (radial distance between visual axis and the center of the pupil) and the pupil center angle (angle between the pupil center plane and corneal surface). Above all, angle kappa has emerged as a useful parameter to consider when planning MIOL implantation. Berdahl and Waring suggested that a MIOL should not be implanted if the preoperative angle kappa is larger than one half the diameter of the central optical region of the multifocal IOL [20]. Fu et al. analyzed 57 eyes of 29 patients undergoing MIOL implantation and concluded that an angle kappa distance greater than 0.5 mm could influence objective visual quality (optical scattering) [21].

In our study sample, mean angle Kappa measurements were 0.37 ± 0.18 mm with the Anterion and 0.26 ± 0.14 mm (*p* < 0.005) with Pentacam, and Bland Altman plots revealed slight dispersion and a small mean difference (0.1 mm ± 0.15). Both devices could be useful in measuring this parameter in clinical practice, but again they should not be considered interchangeable. Likewise, significant differences were found for pupil center angle (Table 1), with a large mean difference (−32.1° ± 115.72) and wide LOAs (−261.2–197.02). On the contrary, pupil center positions X and Y showed excellent agreement and a low mean difference between devices.

Overall excellent agreement was observed for both kerato-metric and pachy-metric measurements. Posterior Kf, Ks and Km differed significantly between devices, but mean differences were small and clinically irrelevant. Several studies have compared the performance of Anterion and other devices [6,7,8,9,10], but only one has provided agreement for posterior corneal surface readings [8]. Overall, for biometric measures, Anterion seems to be more interchangeable with other OCT devices such as CASIA or IOL-Master 700. Tañá-Rivero et al. detected significant differences in WTW measurements taken with the Anterion, IOLMaster 700 (Carl Zeiss Meditec AG, Carl Zeiss Meditec, Jena, Germany) and Pentacam HR, yet concluded that, in clinical terms, Anterion could be considered interchangeable with both these devices [6]. Recently, this group obtained comparable values of keratometry, J0 and J45 vectors, lens thickness, and axial length using the same three devices, but, again, significant differences emerged for anterior chamber depth and central corneal thickness data [7]. Showing the same trend as our results, average posterior corneal power (PCP) and PCP astigmatism were highly repeatable, and agreement was good between the four devices.

Our study has some limitations. While a repeatability analysis was beyond the scope of this study, we only accepted optimal quality images, and all were taken by an experienced ophthalmologist in similar conditions of darkness. While a larger sample would have allowed us to detect more subtle differences, we believe that our sample was sufficiently large to detect clinically meaningful variations. Future research should include an inter-observer analysis in order to improve anterior segment pathologies specificity without reducing the sensitivity [11].

## 5. Conclusions

In summary, corneal power and thickness measurements, except Kf axis, showed excellent agreement between Anterion and Pentacam. Agreement for pupillary position in X and Y vectors *x* and *y* components of the pupil-glint vectors was good, but pupil center distance (angle Kappa), PD and pupil center angle showed poor agreement and, overall, differed significantly between both devices.

By way of overall conclusion, in a clinical setting we would not recommend the interchangeable use of Pentacam and Anterion for measurement of pupil parameters; we would only recommend the interchangeable use of Pentacam and Anterion for corneal measurements.

## Figures and Tables

**Table 1:** Descriptive analysis. Comparison of corneal parameters between the Anterion and Pentacam HR devices.

Parameter	Anterion	Pentacam HR	*p* Value
Anterior Km (D)	43.52 ± 1.54 (40.50–47.25)	42.93 ± 1.52 (40.00–46.20)	0.43
Anterior K2 (D)	44.01 ± 1.64 (40.65–47.81)	43.92 ± 1.59 (41.00–47.70)	0.80
Anterior K1 (D)	43.07 ± 1.56 (40.21–46.71)	42.93 ± 1.52 (40.00–46.20)	0.68
Anterior K1 axis (°)	109.93 ± 62.71 (1.00–180)	76.61 ± 69.64 (0.30–179.90)	0.01 *
Anterior astigmatism (D)	0.93 ± 0.81 (0.05–4.70)	0.98 ± 0.66 (0.00–3.40)	0.77
Anterior K max (D)	44.51 ± 1.72 (40.98–47.56)	44.59 ± 1.76 (41.30–47.40)	0.83
Posterior Km (D)	−6.14 ± 0.27 (−6.94–−5.55)	−6.30 ± 0.28 (−7.00–−5.70)	<0.01 *
Posterior K2 (D)	−6.29 ± 0.30 (−7.38–−5.66)	−6.57 ± 0.86 (−12.00–−5.80)	0.04 *
Posterior K1 (D)	−5.99 ± 0.25 (−6.54–−5.44)	−6.14 ± 0.27 (−6.80–−5.60)	<0.01 *
Posterior K1 axis (°)	124.56 ± 71.69 (0.00–180)	75.87 ± 76.74 (1.10–179.30)	<0.01 *
Posterior astigmatism (D)	−0.30 ± 0.13 (−0.84–−0.04)	0.32 ± 0.13 (0.00–0.60)	0.61
CCT (µm)	546.41 ± 32 (474–615)	552.17 ± 38.05 (479–702)	0.43
TPT (µm)	541.52 ± 31.91 (472–613)	547.06 ± 36.49 (477–674)	0.44
TPP X (mm)	−0.49 ± 0.63 (−1.64–1.04)	−0.38 ± 0.49 (−1.06–0.95)	0.37
TPP Y (mm)	−0.37 ± 0.35 (−1.43–0.44)	−0.33 ± 0.28 (−1.31–0.37)	0.47
Pupil diameter (mm)	5.74 ± 1.34 (2.90–7.80)	3.82 ± 1.60 (1.66–7.06)	<0.01 *
PCP (mm) angle kappa	0.37 ± 0.18 (0.07–1.05)	0.26 ± 0.14 (0.05–0.71)	0.003 *
PCA (°, degrees)	162.89 ± 69.38 (3.00–347)	194.99 ± 78.87 (18.90–359)	0.04 *
PCP X (mm)	−0.22 ± 0.28 (−0.89–0.57)	−0.13 ± 0.23 (−0.63–0.46)	0.12
PCP Y (mm)	0.04 ± 0.20 (−0.55–0.48)	−0.01 ± −0.14 (−0.33–0.29)	0.10

Data were presented as mean ± standard deviation (range) * Statistically significant differences with Student’s *t* test for independent samples. Km = mean keratometry, K1: flat keratometry, K2: steep keratometry, CCT: central corneal thickness, D: diopters TPT: thinnest point thickness, TPP: thinnest point posterior, PCD: pupil center distance, PCA: pupil center angle, PCP: pupil center posterior, µm: microns, mm: millimeters.

**Table 2:** Inter-device measurement agreement between Anterion and Pentacam HR.

Parameter	Difference	95% LoA	*p* Value	CI (95%)
Anterior Km (D)	0.09 ± 0.26	−0.41–0.60	<0.01 *	0.67–0.90
Anterior K2 (D)	0.08 ± 0.41	−0.72–0.89	<0.01 *	0.97–0.99
Anterior K1 (D)	0.13 ± 0.27	−0.39–0.66	<0.01 *	0.98–0.99
Anterior K1 axis (°)	33.32 ± 65.57	−95.19–161.83	<0.01 *	0.41–0.82
Anterior astig. (D)	−0.04 ± 0.45	−0.92–0.84	<0.01 *	0.81–0.94
Anterior K max (D)	−0.07 ± 0.53	−1.13–0.97	<0.01 *	0.95–0.98
Posterior Km (D)	0.16 ± 0.10	−0.03–0.36	<0.01 *	0.93–0.98
Posterior K2 (D)	0.28 ± 0.80	−1.29–1.85	0.051	−0.10–0.66
Posterior K1 (D)	0.15 ± 0.08	−0.01–0.32	<0.01 *	0.94–0.98
Posterior K1 axis (°)	48.69 ± 88.68	−125.12–222.50	0.02 *	−0.00–0.69
Posterior astig. (D)	0.014 ± 0.10	−0.18–0.21	<0.01 *	0.67–0.90
CCT (µm)	−5.76 ± 18.22	−41.47–29.95	<0.01 *	0.87–0.96
TPT (µm)	−5.54 ± 18.08	−40.99–29.91	<0.01 *	0.86–0.95
TPP X (mm)	−0.10 ± 0.35	−0.79–0.58	<0.01 *	0.80–0.94
TPP Y (mm)	−0.04 ± 0.20	−0.44–0.34	<0.01 *	0.80–0.93
Pupil diameter (mm)	1.92 ± 1.16	−0.35–4.20	<0.01 *	0.67–0.90
PCP (mm) angle kappa	0.10 ± 0.15	−0.19–0.40	<0.01 *	0.46–0.83
PCA (°, degrees)	−32.10 ± 115.72	−261.20–197.02	<0.01 *	0.46–0.78
PCP X (mm)	−0.08 ± 0.14	−0.36–0.19	<0.01 *	0.85–0.95
PCP Y (mm)	0.05 ± 0.16	−0.27–0.39	<0.01 *	0.43–0.82

Data were presented as mean ± standard deviation * Statistically significant differences with Student’s *t* test for paired samples. CI: confidence interval, LoA: limits of agreement, Km: mean keratometry, K1: flat keratometry, K2: steep keratometry, CCT: central corneal thickness. TPT: thinnest point thickness, TPP: thinnest point posterior, PCD: pupil center distance, PCA: pupil center angle, PCP: pupil center posterior, D: diopters, µm: microns, mm: millimeters.

## Data Availability

The data presented in this study are available on request from the corresponding author. The data are not publicly available due to the data is part of a research project.
